# Parasitism of Soldiers of the Termite, *Macrotermes gilvus* (Hagen), by the Scuttle Fly, *Megaselia scalaris* (Loew) (Diptera: Phoridae)

**DOI:** 10.3390/insects11050318

**Published:** 2020-05-21

**Authors:** Royto Noknoy, Sakone Sunantaraporn, Atchara Phumee, Padet Siriyasatien, Sunisa Sanguansub

**Affiliations:** 1 Master of Science (Entomology), Department of Entomology, Faculty of Agriculture at Kamphaeng Saen, Kasetsart University, Kamphaeng Saen Campus, Nakhon Pathom 73140, Thailand; kang_39.np@hotmail.com; 2 Vector Biology and Vector Borne Disease Research Unit, Department of Parasitology, Faculty of Medicine, Chulalongkorn University, Bangkok 10330, Thailand; narmspace_open@hotmail.com (S.S.); amphumee@gmail.com (A.P.); padet.s@chula.ac.th (P.S.); 3 Department of Entomology, Faculty of Agriculture at Kamphaeng Saen, Kasetsart University, Kamphaeng Saen Campus, Nakhon Pathom 73140, Thailand

**Keywords:** termite parasite, behavioral changes, parasitic scuttle fly, dipteran larva identification, cytochrome c oxidase I

## Abstract

Termites of the genus *Macrotermes* (Termitidae: Macrotermitinae) are serious agricultural and structural pests, which also play vital roles in ecosystem functioning, and are crucial for the maintenance of tropical biodiversity. They are widely distributed, mainly in Southeast Asian countries; however, the parasitism of termites has been little researched. This research was conducted to identify and study the ecology of the parasitoids of termites at Kasetsart University, Kamphaeng Saen Campus, Nakhon Pathom, Thailand. *Macrotermes gilvus* (Hagen) soldier termites were collected from 25 mounds. In four of the 25 mounds, scuttle fly larvae were found inside the bodies of the soldier termites, and adult flies were found in all of the mounds. Some of the larvae successfully developed to pupae under laboratory conditions. The percentages of parasitized major soldier termites collected from the four mounds were 43.79%, 47.43%, 0.86%, and 3.49%, respectively, and the percentages of parasitized minor soldier termites were 0.64%, 0.00%, 0.21%, and 0.00%, respectively. Larvae, pupae, and adult flies were identified using both morphological and molecular identifications. Molecular identification used the partial nucleotide sequences of the mitochondrial cytochrome c oxidase I (COI) gene. The results of both identification methods identified the parasitic Diptera as the scuttle fly, *Megaselia scalaris* (Loew) (Diptera: Phoridae). The phylogenetic analysis of the 23 scuttle fly samples (11 larvae, 7 pupae, and 5 adults) classified them into two clades: (1) Those closely related to a previous report in India; (2) those related to *M. scalaris* found in Asia and Africa. This is the first discovery of *M. scalaris* in *M. gilvus*. Further investgation into termite parasitism by *M. scalaris* and its possible use in the biological control of termites is needed.

## 1. Introduction

Termites are a group of eusocial insects that are widespread and diverse in tropical and subtropical regions [1]. They can be found everywhere, especially in forests, agricultural areas, and rural and urban ecosystems [2]. In Thailand, four families of termites have been reported: Kalotermitidae, Termopsidae, Rhinotermitidae, and Termitidae [3]. Macrotermitinae is an important subfamily of the Termitidae in Thailand, and includes the genera *Macrotermes*, *Microtermes*, *Ancistrotermes*, *Hypotermes*, and *Odontotermes* [3]. In the genus *Macrotermes* in Macrotermitinae, *Macrotermes gilvus* (Hagen) and *Macrotermes carbonarius* (Hagen) are fungus-growing termites and are commonly found in abundance in Thailand, and elsewhere in Southeast Asia [4]. *Macrotermes gilvus* generally causes damage to agricultural crops, as well as to wooden buildings [5,6]. Several control measures have been used for many years in termite control, especially insecticides. *Macrotermes gilvus* is currently controlled by chemical sprays [5], or by soil treatment with termiticides [6], but termite baits are ineffective [5,6]. However, the use of toxic chemicals can have a serious negative impact on humans and animals. Therefore, biological control or biocontrol techniques are being developed for the alternative control of termites in a safe environment. 

Many studies have demonstrated an association between termites and parasitoid flies. Parasitic flies of the families Phoridae and Calliphoridae have been found in the bodies of soldier termites of *M. gilvus* and *M. carbonarius*, respectively [7,8,9,10,11,12,13,14,15,16,17,18]. Disney et al. (2009) found the larvae of the parasitoid *Misotermes mindeni* (Disney and Neoh) (Diptera: Phoridae) in the heads of *M. gilvus* in Malaysia [10]. This parasitoid caused morphological changes in the termite, including a rounded head capsule and shortened mandibles. It also caused behavioral changes, reducing aggressive behavior in major soldier termites [7]. Recently, two new species of scuttle fly, *Megaselia nigrifinis* (Liu and Chen) and *Megaselia setidifferitatis* (Liu and Chen) were reared from dead termites, *Odontotermes formosanus* (Shiraki) in Nanjing, China [19]. Therefore, it is evident that scuttle flies are associated with termites by feeding on living or dying termites. However, the association between the scuttle flies and termites is poorly understood. Therefore, in this study, we investigated the association between parasitic Diptera and the termite genus *Macrotermes* in an area in central Thailand. The objectives of the study were as follows: (1) to identify the parasitic fly species found in the termite bodies and in the termite mounds; (2) to use molecular techniques to associate larvae and adults of the parasitic fly; (3) to determine whether the parasite changed the morphology and/or behavior of the attacked termites; (4) to determine the variation in rates of parasitism in different termite mounds; (5) to correlate the rates of parasitism with the physical characteristics of the termite mounds and the environments in which they occurred.

## 2. Materials and Methods 

### 2.1. Termite Collection 

The termites of the genus *Macrotermes* [20,21] are found commonly in Kasetsart University, Kamphaeng Saen Campus (14°01Ȳ16.08″ N, 99°58′53.63″ E), Nakhon Pathom, Thailand. *Macrotermes* termites were collected from 25 mounds from August 2018 to August 2019. The mounds occurred among groves of trees, near reservoirs and irrigation canals, and in vegetable plots, orchards, parks, human activity areas, and buildings. We collected 50–300 major and 100–500 minor soldier termites per mound, depending on the mound size and the abundance of termites in each mound. The termite mounds were excavated from the side, and soldier termites were removed into containers. In two mounds, which were partly destroyed before our survey, we dug around the base of mound. Further and more detailed investigations were carried out on four mounds in which termites parasitized by phorid flies were collected. 

### 2.2. Aggressive Behavior

The aggressive behavior of the soldier termites was investigated in the soldiers collected from each mound. Major and minor soldiers were tested separately. The termites were placed individually in a petri dish and a pair of forceps was used to disturb them. A retaliatory response (fighting or biting) to the forceps, or its absence, was recorded. 

### 2.3. Parasitic Diptera Collection and Morphological Identification

The parasitic Diptera were collected from four termite mounds, including 240 larvae found by incising the termites’ head capsules, or which hatched from dead termites’ abdomens. Of these, 67 live larvae were kept in glass bottles and covered with an organdy cloth at room temperature for pupation, and the rest were preserved in 70% ethanol. The specimens were identified by taxonomic family using their morphological features: larva [22,23,24], pupa [23,24], and adult [23,24,25,26,27]. Adult flies were identified by genus and species by R.H.L. Disney. The parasitized soldier termites were compared with the parasitized soldier termites of the reports, which showed their morphological changes [7,11,16]. The characteristics and environments of the parasitized mounds were noted, relative to the non-parasitized mounds.

### 2.4. Molecular Identification of Parasitic Diptera

#### 2.4.1. DNA Extraction

Genomic DNA was extracted from individual specimens (11 larvae, 7 pupae, and 5 adults) by using an Invisorb^®^ Spin Tissue Mini Kit (STRASTEC Molecular GmbH, Berlin, Germany), according to the manufacturer’s instructions. The extracted DNA was kept at −20 °C for long-term storage.

#### 2.4.2. PCR Amplification

A semi-nested polymerase chain reaction (semi-nested PCR) was used for amplifying the mitochondrial cytochrome c oxidase I (COI) gene twice. The oligonucleotide primers were designed, based on the COI sequences of the Phorid fly from the reports [24,27,28], as the forward primer 5′-GGTCAACAAATCATAAAGATATTGG-3′ and reverse primer 5′-TAGACTTCTGGGTGGCCAAAGAATCA-3′ for the first amplification. The reverse primer that was used for the second amplification was newly designed based on the COI gene sequences of *Megaselia scalaris* obtained from the GenBank database as 5′-AATGATGTATTAAAATTTCG-3′. The primers were synthesized by Integrated DNA Technologies, Inc. (IDT) (Singapore Science Park II, Singapore). PCR amplifications were performed in a final volume of 25 μL, containing 10X *Taq* buffer, 2.5 mM MgCl_2_, 2.5 mM dNTP, 10 μM of forward and reverse primers, one unit of *Taq* DNA polymerase (Thermo Scientific, Waltham, MA, USA), and 3 μL of DNA template. The PCR was performed under thermal cycling profiles with an initial denaturation at 95 °C for 3 min, and the 35 cycles of amplification were as follows: denaturation at 95 °C for 1 min; annealing at 57 °C for 1 min for the first and second PCRs; extension at 72 °C kept together with units for 1.5 min; final extension at 72 °C for 7 min. The PCR products were separated by size by 1.5% agarose gel electrophoresis. The specific PCR product sizes were photographed using Quantity One quantification analysis software, version 4.5.2 (Gel DocEQ System; Bio-Rad, Hercules, CA, USA).

#### 2.4.3. DNA Cloning and Sequencing

The positive PCR amplicons were ligated into a pGEM-T easy vector (Promega, Madison, WI, USA) using a Rapid DNA ligation kit (Promega), according to the manufacturer’s instructions. The recombinant DNA was transformed into the *Escherichia coli* DH5α competent cell and screened using the blue-white colony screening system. The positive colonies with an inserted COI gene were cultured on Luria–Bertani agar. The plasmid DNA containing the relevant DNA was extracted using an Invisorb Spin Plasmid Mini Kit (STRASTEC Molecular GmbH, Berlin, Germany), following the manufacturer’s instructions. The extracted plasmid was sequenced by the commercial service of Macrogen Inc., South Korea, using a universal forward T7 primer. 

#### 2.4.4. Phylogenetic Analysis 

The obtained nucleotide sequences were analyzed using BioEdit, a program to edit the alignment of sequences, version 7.1.9 [29]. The edited nucleotide sequences were compared with the nucleotide sequences in the GenBank database using BLASTN searches (http://www.ncbi.nlm.nih.gov), and all of the nucleotide sequences from this study were submitted to the GenBank database using Banklt (https://www.ncbi.nlm.nih.gov/WebSub/). The percentages of intraspecific variations were analyzed using BioEdit 7.1.9. A phylogenetic tree was constructed using the maximum likelihood method (ML) with the general time reversible model (GTR) and bootstrap analysis with 1000 replications in Molecular Evolutionary Genetics Analysis, version X (MEGAX) [30].

## 3. Results

### 3.1. Morphological Identification of Termites 

In all of the 25 mounds in the various environments, the termite species present was identified as *Macrotermes gilvus* (Hagen). The species was identified using the major soldier caste, according to Ahmad (1965) [20] and Intanai (1987) [21]. Soldiers, which were later shown to be parasitized, exhibited no abnormalities or changes in morphology, such as a rounded head capsule or mandibles of reduced size. 

### 3.2. Aggressive Behavior

In our tests, the aggressive soldier termites responded to disturbances made by a pair of forceps by fighting, seizing, and biting, while the non-aggressive soldier termites did not show this behavior, but moved slowly and with little vigour. We found that all of the parasitized soldier termites, and some of the non-parasitized soldier termites from parasitized mounds, were non-aggressive. The results of the experiments on aggressive behavior for the four mounds and the two castes of soldiers are shown in Table 1. 

### 3.3. Parasitic Diptera

**Figure 1:**
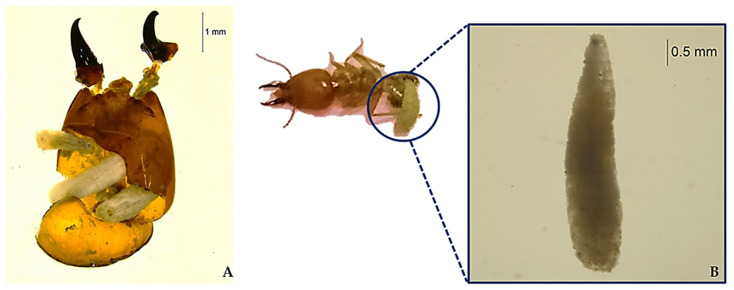
Larvae in the soldier termite head (A) and emerging from the soldier termite body (B).

A total of 240 larval stages were found in four mounds, divided into the 14 dead larvae found in the heads, and 226 living larvae found in the bodies of the *M. gilvus* soldiers from the four mounds, as shown in Figure 1A,B, respectively. Only one phorid larva was found in an individual soldier termite, with a single exception in which five larvae were found in the head of one soldier. Sixty-seven pupae developed from the larvae under laboratory conditions, but adult flies did not emerge from them. However, 75 adult Phorid flies were found in the mounds. They showed the rapid bursts and short pauses of movement that are characteristic of the family Phoridae [31]. The specimens were preserved in the museum of the Entomology Department, Kasetsart University, Kamphaeng Saen. The numbers and percentages of parasitized soldiers of the two castes for the four mounds are shown in Table 1.

### 3.4. Morphological Identification of Parasitic Diptera

All stages of the fly were identified and confirmed by R.H.L. Disney as the scuttle fly, *Megaselia scalaris* (Loew). 

### 3.5. Molecular Identification of Parasitic Diptera

**Figure 2:**
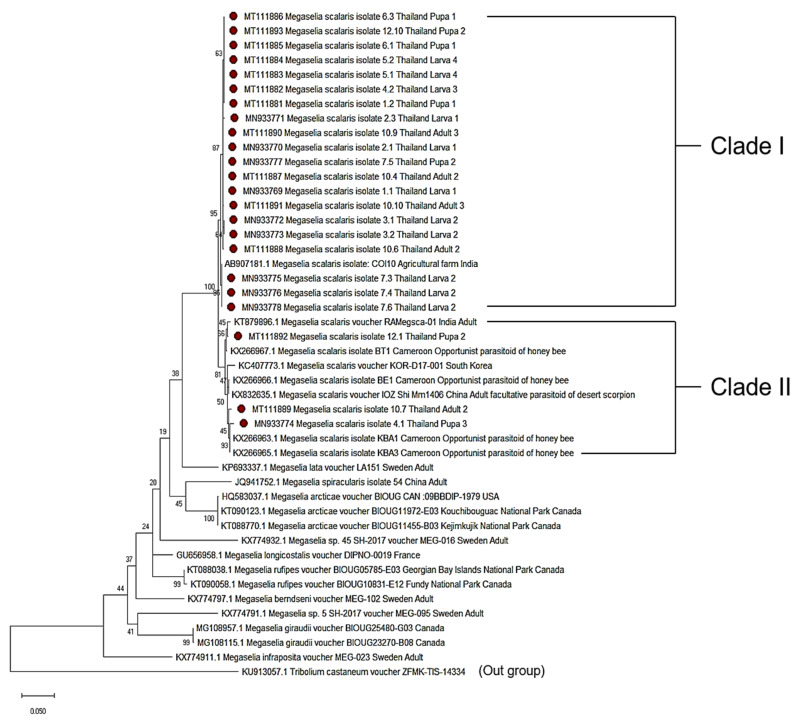
Phylogenetic tree of parasitic Diptera constructed from partial c oxidase I (COI) sequences. The maximum likelihood method was based on the general time reversible model and 1000 bootstrap replications. The sequences from this study, indicated in red, were compared with the reference sequences obtained from GenBank.

A total of 23 parasitic Diptera specimens (11 larvae, 7 pupae, and 5 adults) were identified by using the molecular technique. The partial nucleotide sequence of the COI gene by PCR was 634 bp. The BLAST results for all of the specimens showed that the scuttle fly, *M. scalaris*, had 98.08–99.84% similarity to the NCBI database. All sequence samples were submitted to the GenBank and assigned the accession numbers of MN933769–MN933778 and MT111881–MT111893. The percentages of intraspecific variation was 0.0–3.2%. Specimens were similar to a lineage of *Megaselia scalaris* from China with 97.51–99.20% similarity, and to a lineage of the same species from Cameroon with 97.87–99.39% similarity. The specimens could be classified into two clades, as shown in Figure 2. The first clade was related to *M. scalaris* found in an agricultural farm in Tamil Nadu, India (AB907181). The second clade was closely related to previous reports from India (KT879896), South Korea (KC407773), Cameroon, which concerned an opportunist parasitoid of honey bees (KX266963, KX266965, KX266966, and KX266967), and a report from China, which concerned a facultative parasitoid of the desert scorpion (KX832635), as shown in Figure 2. *Tribolium castaneum*, accession no. KU913057, was used as an outgroup.

### 3.6. Characteristics of Parasitized Termite Mounds

We investigated the characteristics of the four parasitized mounds. One mound was very tall and large, two mounds had been destroyed previously, but their origins were quite large, and the fourth was quite low and small. Every parasitized mound was located near a water source with high humidity, moist soil, and low ambient light intensity, and they were overgrown with grass and weeds.

## 4. Discussion

This study is the first record of *Megaselia scalaris* as a parasite of termites. The species is normally saprophagous, feeding on both plant and animal dead matter, but has been recorded scavenging in the nests of social insects, including *Macrotermes gilvus* [7], and feeding on stored food, mushrooms, and living plants [11]. It has also been recorded as a parasite or parasitoid of various species of Arthropoda [28,31,32,33,34,35,36,37,38,39,40,41,42], and Mollusca [42]. Both major and minor soldiers were parasitized, but few minor soldiers were attacked, as shown in Table 1. This is the first record of phorid attacks on minor soldiers of termites. The parasitized soldiers showed a normal morphology, in contrast to earlier reports [7,11,16], in which the soldiers of *M. gilvus,* which were attacked by phorids of the species *Misotermes mindeni*, had rounded rather than oblong head capsules, and mandibles of reduced size. The parasitized soldiers did not show aggressive behavior, as shown in Table 1, which is similar to the reports of Neoh and Lee [7] on *Macrotermes gilvus* attacked by the phorid *Misotermes mindeni*. There is clearly much variation in the percentages of parasitism in the different termite mounds, as shown in Table 1, and most of the mounds did not contain parasitized soldiers. Some of the variation in the percentages of parasitism may be linked to the environment in which the termite mounds occurred. The parasitized mounds were all located in humid and shaded habitats, and the phorids appeared to have a preference for this type of environment. The phorid *M. mindeni* was also found to attack *M. gilvus* mounds in similar humid habitats [7,14]. 

Phorid flies have been suggested as the biocontrol agents of many pests. The ant-decapitating fly (genus *Pseudacteon*) has been suggested for the control of fire ants (genus *Solenopsis*) [43,44,45]. *Megaselia scalaris* has been suggested for the control of the cattle tick, *Rhipicephalus* (*Boophilus*) *microplus* (Canestrini) [42,46]. Miranda-Miranda et al. (2010) reported the success of *M. scalaris* culture on the tick *R. microplus*, and proposed the idea of breeding *M. scalaris* strains under laboratory conditions to estimate the possibility of inducing them to be host-specific [42]. *Misotermes mindeni* has been suggested for the control of the termite, *M. gilvus* [14]. In this study, *M. scalaris* was detected in a low percentage of the parasitized soldier termites of each mound under natural conditions, which were similar to those in which *M. mindeni* was found. The reports of parasitic flies, including *M. scalaris* in the desert scorpion, and *Mesobuthus eupeus mongolicus* [28] and *M. mindeni* in the termite, *M. gilvus* [7,11,12,13,14], suggest the possibility of studying the relationship between flies and their hosts under laboratory conditions in order to understand their life cycle, the mechanism of parasitization, the influence of the flies on their host, and their mode of life within the host’s body. Further studies should investigate the parasitism of *M. scalaris* within major soldiers or other castes of termites under laboratory conditions for eventual application as a biocontrol agent of *Macrotermes* or other termites. 

To the best of our knowledge, this study is the first report relating to the identification of the different developmental stages (larva, pupa, and adults) of the phorid fly parasite of termites with a molecular technique. The morphological identification of the immature stages of a genus or species is often impossible. Therefore, genetic techniques, especially the DNA barcoding method, can play an important role in improving the accuracy and efficiency of the identification of parasitic species and their diversity. From the comparison of the specimen COI gene with GenBank, our *M. scalaris* specimens are more similar to the *M. scalaris* with parasitoid behavior than to the *M. scalaris* with normal (saprophage) behavior. Our phylogenetic analysis revealed that the 23 samples which showed the *M. scalaris* in this study were classified into two clades: the Thailand clade, related to *M. scalaris* found in the agricultural farm at Tamil-Nadu, India (AB907181), and the other clade, closely related to previous reports from India, South Korea, Cameroon, and China. However, because of limited information on the molecular evolution as well as the sequence data for *M. scalaris* in Thailand and Southeast Asia, we are not able to compare our results with other studies within the country and region. The molecular studies could be used for the classification and determination of the genetic variations in *M. scalaris*, and our report provides fundamental data for further evolutionary and ecological studies of *M. scalaris* in Thailand. Therefore, extensive surveys and more precise studies of *M. scalaris* in Thailand, covering more areas and larger sample sizes, must be performed in order to understand its geographic location, evolution, and ecology interaction. 

## 5. Conclusions

A parasitic dipteran of the termite genus *Macrotermes* was identified as the scuttle fly, *Megaselia scalaris* based on morphological identification and molecular analysis using the mitochondrial COI gene. We show that: (1) the scuttle fly *M. scalaris* is a parasite of soldiers of the termite *M. gilvus*; (2) the parasitized soldier termites show behavioral changes but do not show morphological changes; (3) both the adult and immature stages of the parasitic Diptera of termites can be identified using molecular techniques, and the molecular identification of *M. scalaris* in Thailand and Southeast Asia is possible using a newly designed reverse primer. 

## Figures and Tables

**Table 1:** Numbers and percentages of major and minor soldiers in four *Macrotermes gilvus* mounds that were (a) non-aggressive, and (b) both non-aggressive and parasitized.

Mound Number	Caste of Soldier Termites	No. of Individual Soldiers	No. of Non-Aggressive Soldiers	No. of Parasitized Soldiers	Percentage of Soldiers	
					Non-Aggressive	Parasitized
1	Major	153	73	67	47.71	43.79
	Minor	312	4	2	1.28	0.64
2	Major	331	317	157	95.77	47.43
	Minor	515	379	0	73.59	0.00
3	Major	350	11	3	3.14	0.86
	Minor	489	3	1	0.62	0.21
4	Major	172	45	6	26.16	3.49
	Minor	356	0	0	0.00	0.00
